# Pestiviruses infection: Interferon-virus mutual regulation

**DOI:** 10.3389/fcimb.2023.1146394

**Published:** 2023-03-02

**Authors:** Tianqi Hong, Yi Yang, Pengzhi Wang, Guoqiang Zhu, Congrui Zhu

**Affiliations:** ^1^ College of Veterinary Medicine, Yangzhou University, Yangzhou, China; ^2^ Jiangsu Co-Innovation Center for Prevention and Control of Important Animal Infectious Diseases and Zoonoses, Yangzhou University, Yangzhou, China; ^3^ Joint Laboratory of International Cooperation on Prevention and Control Technology of Important Animal Diseases and Zoonoses of Jiangsu Higher Education Institutions, Yangzhou University, Yangzhou, China; ^4^ Joint International Research Laboratory of Agriculture and Agri-Product Safety of Ministry of Education of China, Yangzhou University, Yangzhou, China; ^5^ College of Animal Science, South China Agricultural University, Guangzhou, China

**Keywords:** interferons, antiviral, pestiviruses, antagonism, interaction

## Abstract

Pestiviruses are a class of viruses that in some cases can cause persistent infection of the host, thus posing a threat to the livestock industry. Interferons (IFNs) are a group of secreted proteins that play a crucial role in antiviral defense. In this review, on the one hand, we elaborate on how pestiviruses are recognized by the host retinoic acid-inducible gene-I (RIG-I), melanoma-differentiation-associated protein 5 (MDA5), and Toll-like receptor 3 (TLR3) proteins to induce the synthesis of IFNs. On the other hand, we focus on reviewing how pestiviruses antagonize the production of IFNs utilizing various strategies mediated by self-encoded proteins, such as the structural envelope protein (E^rns^) and non-structural protein (N^pro^). Hence, the IFN signal transduction pathway induced by pestiviruses infection and the process of pestiviruses blockade on the production of IFNs intertwines into an intricate regulatory network. By reviewing the interaction between IFN and pestiviruses (based on studies on BVDV and CSFV), we expect to provide a theoretical basis and reference for a better understanding of the mechanisms of induction and evasion of the innate immune response during infection with these viruses.

## 1 Introduction

Pestiviruses are a group of enveloped positive-sense single-stranded RNA ((+) ssRNA) viruses, which can spread horizontally and vertically between hosts. Pestiviruses have a wide spectrum of hosts, causing disease in many animals, such as pigs, cattle, sheep, goats, pronghorns, giraffes, *Phocoena phocoena*, etc. (Vilcek & Nettleton, 2006; Jo et al., 2019). For a long time, the genus *Pestivirus* has been recognized as containing four species: *Bovine viral diarrhea virus 1* (BVDV-1), BVDV-2, *Classical swine fever virus* (CSFV), and *Border disease virus* (BDV) (Simmonds et al., 2017; Yesilbag et al., 2017). Until 2017, the revised version of the International Committee on Taxonomy of Viruses (ICTV) taxonomy classified the genus *Pestivirus* into 11 species (from *Pestivirus A* to *Pestivirus K*) (Smith et al., 2017). BVDV is a representative species of pestiviruses. Most of the cattle infected with BVDV have mild symptoms or only present subclinical symptoms, and some produce a series of clinical symptoms that cause multiple systemic diseases, affecting digestive tract (diarrhea), respiratory tract (cough), and reproductive tract (reproductive disorder) (Walz et al., 2010; Tautz et al., 2015; Sozzi et al., 2020). Among them, about 2% develop into persistent infection (PI) cattle (Schweizer & Peterhans, 2014; Deng et al., 2020). Those subsets of cattle have no obvious clinical symptoms and do not generate BVDV-specific antibodies, but carry and shed viruses for their whole life. Therefore, PI cattle are the main virus reservoirs and cause a serious threat to healthy cattle (Klimowicz-Bodys et al., 2022). According to whether it causes cytopathic lesions, BVDV can be classified into cytopathic (CP) biotype and non-cytopathic (NCP) biotype (Gillespie et al., 1961). CSFV is another important representative species of pestiviruses, which causes acute contact infectious disease in swine. CSFV is characterized by high fever, hemorrhages of skin and inner organs, along with respiratory and gastrointestinal syndromes, bringing significant economic losses to the pig industry. As typical viruses of the genus *Pestivirus*, BVDV and CSFV have been studied extensively and deeply. Hence, these two viruses are used as examples to expound some characteristics of the interplay between pestiviruses and the host in the following.

**Figure 1:**
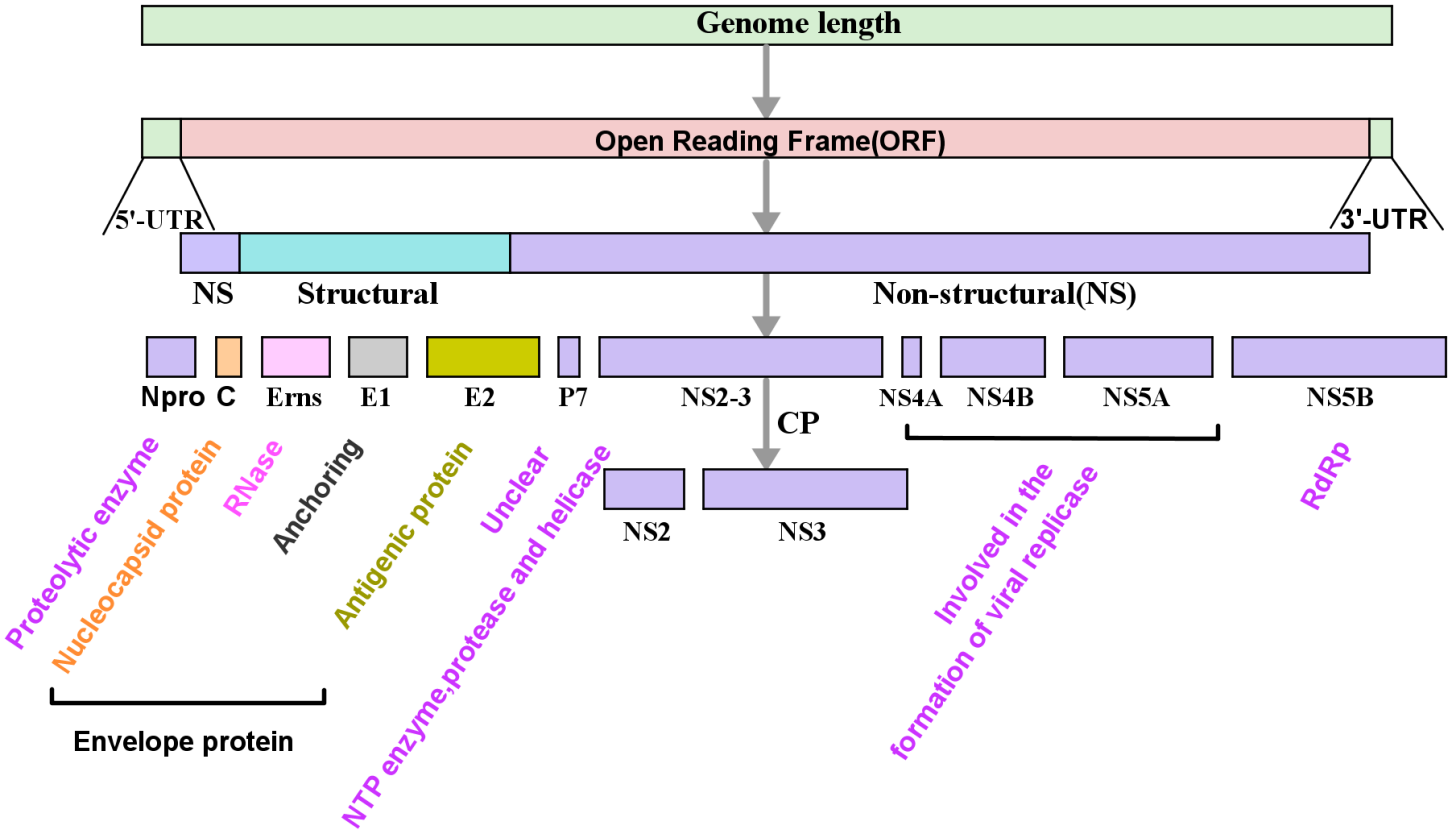
Schematic diagram of the genome and its encoded protein of pestiviruses. The genome of *Pestivirus* consists of the 5’-untranslated regions (5’-UTR), a large open reading frame (ORF), and the 3’-untranslated regions (3’-UTR). The ORF is translated into a polyprotein which is further cleaved by protease into multiple viral proteins, including four structural proteins, viral capsid protein **(C)**, envelope proteins (E^rns^, E1, and E2), and eight non-structural proteins (N^pro^, p7, NS2, NS3, NS4A, NS4B, NS5A, and NS5B). In the picture, the color text is a description of the function of the corresponding protein. RdRp, RNA-dependent RNA polymerase.

The genome of *Pestivirus* viruses is approximately 12.3 kb, which can encode at least 11 viral proteins (**Figure 1**) (Neill et al., 2019; Johnston et al., 2020; Walz et al., 2020). N^pro^ is a non-structural protein unique to pestivirus with autoprotease activity and is often used for classifying pestiviruses. In addition, E^rns^ is also a specific protein for the genus *Pestivirus*, which usually exists in the form of homodimers linked by disulfide bonds. E^rns^ is a highly conserved membrane protein with ribonuclease (RNase) activity. These two proteins play a significant role in antagonizing the innate immune of the host (Peterhans & Schweizer, 2010). Studies have demonstrated that N^pro^ and E^rns^ were related to the formation of PI.

Interferons (IFNs) are a class of glycoproteins with multi-biological activities, containing three families (type I, type II, and type III). The antiviral response mediated by IFNs is a vital way of the host defense against viral infections (Fensterl et al., 2015). Studies have shown that IFNs can dampen the replication of different biotypes and genotypes of BVDV (Elsheikh et al., 2019; Quintana et al., 2020a).

When the pestiviruses infect susceptible animals, it can cause the animals to produce corresponding IFNs to resist the invasion of the pestiviruses (Husser et al., 2011; Dong et al., 2013; Reid et al., 2016; Cai et al., 2017; Maldonado et al., 2020; Li et al., 2021). Indeed, some subsets of pathogenic microorganisms have evolved numerous strategies to escape the host’s immune response to survive during infection, which will facilitate the replication and transmission of viruses *in vivo* (Garcia-Sastre, 2017; Segredo-Otero & Sanjuan, 2020). Many researchers have found that pestiviruses could use their self-encoded proteins to antagonize the antiviral impact of the host by inhibiting IFNs, antagonizing apoptosis, regulating mitophagy, and other ways (Jefferson et al., 2014; Gou et al., 2017; Dong et al., 2018; Zhang et al., 2018; Cao et al., 2019b; Hardy et al., 2020; Fan et al., 2021; Xie et al., 2021; Ma et al., 2022). Among them, the research on the inhibitory effects of N^pro^ and E^rns^ proteins on IFNs is relatively deep but lacks a systematic summary at present. Based on this, we will give a comprehensive review as possible in this article.

In this review, we introduced the inhibitory effect of IFNs on pestiviruses in the following aspects: a) Pestiviruses infection stimulates the production of IFNs; b) IFNs induced interferon-stimulated genes (ISGs) to exert anti-pestivirus activities. What’s more, we focus on reviewing the latest advances in pestivirus structural proteins (E^rns^) and non-structural proteins (N^pro^) in antagonizing IFN. Viewing that the biological functions of IFN-II are mainly in pro-inflammatory and immune regulation, which has limited direct antiviral activity (Walker et al., 2021), and the induction pathway of IFN-II is disparate from IFN-I and IFN-III. Thus, we will focus on IFN-I and IFN-III to elaborate on the complex interplay between pestiviruses and IFNs.

## 2 The production of interferons induction by pestiviruses infection

### 2.1 Sensing complexes during pestiviruses infection

Cao et al. showed that CSFV infection could regulate the expression of Toll-like receptors (TLRs). *In vitro* infection trials showed that the C strain and Shimen strain could promote the expression of TLR2, TLR4, and TLR7, but there was no impact (C strain) or inhibition (Shimen) on the production of TLR3 (Cao et al., 2015). Similarly, BVDV infection also regulates the differential expression of *TLRs* genes in bovine peripheral blood mononuclear cells (PBMCs) (Lee et al., 2008). Overall, studies have demonstrated that pestiviruses infection affected the innate immune response by regulating TLRs (Cao et al., 2019a). In addition, Hüsser et al. indicated that when PK-15 cells of which retinoic acid-inducible gene-I (RIG-I), melanoma-differentiation-associated gene 5 (MDA5), and TLR3 were knocked down and infected with CSFV respectively, the bioactivity level of IFN-I was observably decreased, and its reduction degree was almost consistent with the degree of RIG-I, MDA5, and TLR3. The results suggested that RIG-I, MDA5, and TLR3 were sensors to recognize CSFV infection and played a crucial role in CSFV infection (Husser et al., 2011).

### 2.2 Activation of interferons response by pestiviruses infection

**Figure 2:**
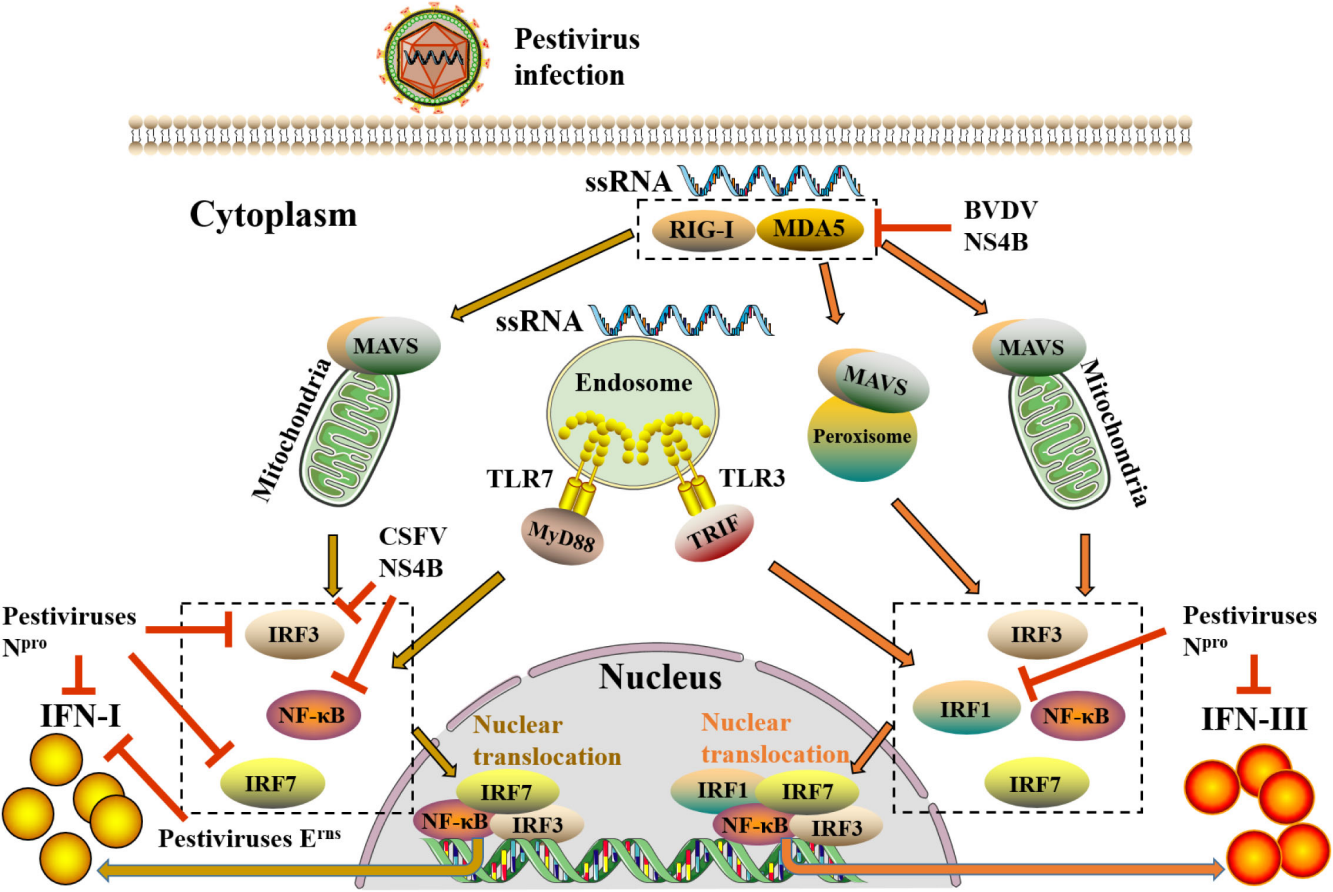
The induction pathways of IFN-I and IFN-III after pestivirus infection and evasion of pestiviruses to IFN-I and IFN-III. When pestiviruses infect host cells, they are recognized by RIG-I-like receptors (RLRs) and TLRs. RLRs (RIG-1 and MDA5) *via* the activate of mitochondrial antiviral signaling protein (MAVS) on mitochondria and peroxisomes, whereas TLRs (TLR3 and TLR7) through activating TIR domain-containing adapter-inducing interferon-β (TRIF) and MyD88 on endosomes, transmit upstream receptor molecular signals downstream, thereby activating downstream transcription factors, including IRFs and NF-κB. Subsequently, these transcription factors are phosphorylated and transferred to the nucleus, where they bind to the upstream promoter regions of IFN-I and IFN-III genes to initiate the expression of IFN-I and IFN-III. Different from the induction of IFN-I, peroxisome-MAVS is involved in the induction of IFN-III. During IFN-III induction, peroxisome-MAVS is the primary downstream adaptor molecule of the RLRs pathway, and mitochondria-MAVS only participate in a small amount of IFN-III induction. The abduction of IFN-I requires an enhanceosome which is composed of highly coordinated and cooperative NF-κB, IRF3, and IRF7. However, in addition to transcription factors NF-κB, IRF3, and IRF7, the involvement of IRF1 is also required for IFN-III. Moreover, pestiviruses can also antagonize the production of IFN-I and IFN-III through multiple self-encoded proteins. In the figure, the yellow arrow shows the induction pathway of IFN-I, and the orange arrow shows the induction pathway of IFN-III, whereas the red line indicates the inhibition of pestiviruses.

Pestiviruses infected cells initiate a variety of antiviral responses to resist the invasion of pestiviruses, in which IFNs play a crucial role in dampening pestiviruses replication. During pestivirus infection, viruses are recognized by pattern recognition receptors (PRRs) of the host to activate interferon regulatory factors (IRFs) and nuclear factor-kappa B (NF-κB) and then induce the synthesis of IFNs (**Figure 2**).

Early studies have shown that CSFV infection was recognized by pattern recognition receptors RIG-1, MDA5, and TLR3 (mainly RIG-I), which activated downstream signaling molecules MAVS, IRF3, and NF-κB, leading to the production of IFN-α/β and a large number of inflammatory cytokines (TNF-α, IL-1β, and IL-6) (Husser et al., 2011; Dong et al., 2013). NS5A, a nonstructural protein of CSFV, promoted the synthesis of IFN-α by up-regulating the activation of myeloid differentiation factor 88 (MyD88) and IRF7 (Cao et al., 2019b). Moreover, the hemoglobin subunit beta of host protein specifically interacted with the capsid protein (C) of CSFV to trigger IFNs signal transduction through the RIG-I pathway (Li et al., 2013). Furthermore, recently, researchers discovered that CP BVDV-1 infection MDBK cells could promote the synthesis of IFN-β by inducing the expression and nuclear translocation of NF-κB, IRF1, and IRF7 (Maldonado et al., 2020).

Although many previous works have elucidated the induction of IFN-I by pestiviruses infection, there was few reports have addressed the production and mechanism of IFN-III. Reid et al. reported the induct of IFN-III by pestiviruses infection for the first time, demonstrating that BVDV-infected bovine pDC could induce IFN-I and IFN-III with different kinetics, and the IFN-III had the characteristics of acid-labile (Reid et al., 2016). Furthermore, the major component of total IFNs induced by BVDV during *in vivo* and *in vitro* infection was IFN-III (Reid et al., 2016). IFN-I and IFN-III were equivalent in terms of transcription level in the above studies, but the expression of IFN-I protein was lower than IFN-III. We hypothesize that there may be post-transcriptional regulation, leading to a quite difference in the expression of IFN-I and IFN-III. Subsequently, studies found that the *in vivo* and *in vitro* infection of CSFV could induce the expression of IFN-III and several key ISGs through the activation of signal transducer and activator of transcription 1 (STAT1) and NF-κB (Reid et al., 2016; Cai et al., 2017). Intriguingly, although CSFV infection could induce IFN-III production, the up-regulation of IFN-III *in vivo* and *in vitro* was limited. According to the research on the exploration of virus escape from host immune response in recent years, the hypothesis is raised whether pestiviruses can also restrain the up-regulated level of IFNs *via* a certain mechanism, thereby antagonizing the antiviral response of the host (Garcia-Sastre, 2017; Segredo-Otero & Sanjuan, 2020).

## 3 Interferons-induced IFN-stimulated genes to defend against pestiviruses infection

### 3.1 Antiviral effects of interferons on pestiviruses

The antiviral activities of IFNs have been extensively studied on a variety of viruses, including pestiviruses. *In vitro* experiments showed that exogenous human IFN-α could inhibit the replication of BVDV, and there was no distinguished difference between CP BVDV genotypes (Elsheikh et al., 2019). Quintana et al. used the non-structural protein NS3 established direct high-throughput Cell-ELISA and demonstrated that IFN-I and IFN-III had antiviral effects against different biotypes and genotypes of BVDV, and the suppression of CP BVDV was much more pronounced than the NCP BVDV, whereas the NCP BVDV-2 was more sensitive to IFN-I (Quintana et al., 2018). In addition, both ovis aries IFN-ϵ and feline IFN-ω have typical characteristics of IFN-I and showed antiviral activity against BVDV (Guo et al., 2020; Wang et al., 2020).

The *in vivo* trials illustrated that IFN-III could protect cattle from BVDV infection (Quintana et al., 2020b). Additionally, some researchers successfully established the infection model of BALB/c mice using the NCP BVDV-2 virulent strain and attenuated strain and used this model to evaluate the impacts of IFN-I and IFN-III in the prevention and treatment of BVDV infection (Quintana et al., 2020a). It discovered that both IFN-I and IFN-III could reduce BVDV infection *in vivo*, and IFN-III was more effective in preventing BVDV infection, while IFN-I had better performance in terms of treatment (Elsheikh et al., 2019; Quintana et al., 2020a). The above results indicated that, like endogenously produced IFNs, exogenous IFNs also have antiviral activity *in vitro* and *in vivo*, and their inhibitory roles on CP BVDV are more pronounced.

### 3.2 The mechanism of interferons defense against pestiviruses

**Figure 3:**
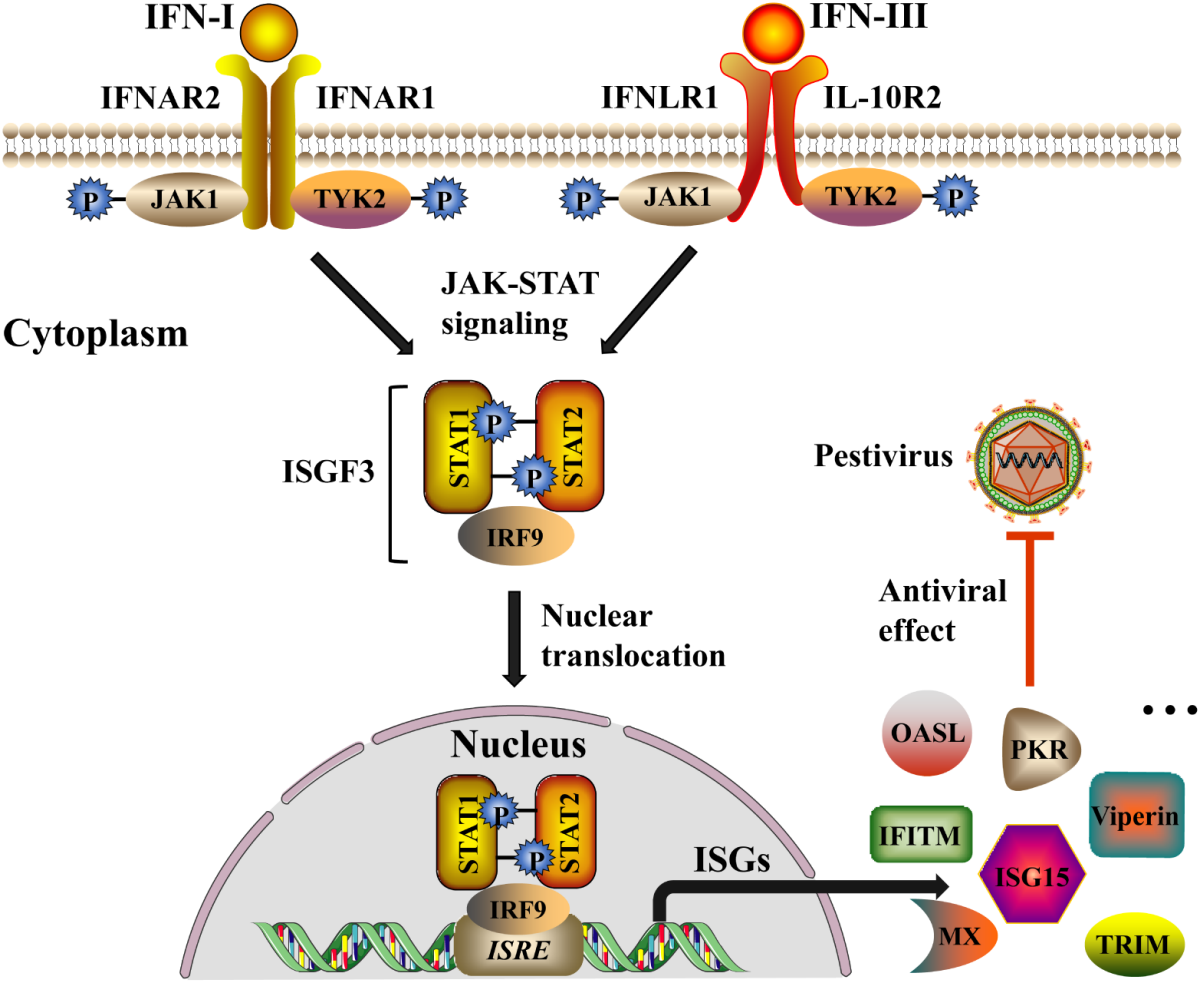
IFN-I and IFN-III induce signaling cascades to inhibit pestivirus infection. Both IFN-I and IFN-III trigger a series of signaling cascades by activating the JAK-STAT signaling pathway. IFN-I and IFN-III respectively bind to the heterodimeric receptors IFNAR (IFNAR1 and IFNAR2) and IFNLR (IFNLR1 and IL-10R2) on the cell membrane, causing the conformation change of the intracellular part of the receptor subunit, and then leads to phosphorylation of JAK and tyrosine kinase 2 (TYK2). Activation of JAK and TYK2 allows STAT1 and STAT2 to be recruited and phosphorylated, and the phosphorylated STAT1 and STAT2 further recruit IRF9 to form interferon-stimulated gene factor 3 (ISGF3). Subsequently, ISGF3 translocates from the cytoplasm to the nucleus and binds to ISRE sequences of the ISG promoter region to initiate the expression of a variety of ISGs (for example, Mx, TRIM, IFITMs, ISG15, PKR, OASL, Viperin, etc.), and ultimately inhibit the replication and spread of the pestiviruses. The black arrow indicates the induction pathway of ISGs, and the red line indicates the inhibition of pestiviruses.

In reality, IFNs do not directly inactivate viruses but rather synthesize antiviral proteins to dampen the replication of the viruses. Lately, there have been ongoing and increasing studies found that the signal transduction mechanism of IFN-I and IFN-III were similar (Onoguchi et al., 2007; Sommereyns et al., 2008; Mordstein et al., 2010), both of which through the Janus kinase (JAK)-signal transducer and STAT signaling pathway induces the expression of ISGs, thus exerting a synergistic antiviral response (**Figure 3**) (Forero et al., 2019). Depending on the difference in working principle, ISGs could be classified into three types: inhibit virus entry (Mx, TRIM, IFITMs), inhibit protein synthesis (ISG15, PKR, OASL), and inhibit virus release (Viperin) (Raftery & Stevenson, 2017). In this part, we overview some ISGs related to the suppression of pestiviruses.

#### 3.2.1 Mx

Myxovirus resistance (Mx) protein was one of the ISGs composed of Mx1 and Mx2 protein (also known as MxA and MxB in humans) (Holzinger et al., 2007). In the cytoplasm, Mx1 acted in the early period of the virus replication cycle and targeted the viral nucleoprotein through the GTPase activity of the N-terminal GTPase domain (Haller & Kochs, 2011). Mx2 was discovered later than Mx1 and has adopted the function of specifically restraining capsid entry into the nucleus (Schneider et al., 2014). He et al. used the exogenously expressed Mx1 fusion protein to treat CSFV-infected cells and found that Mx1 effectively inhibited CSFV replication in a dose-dependent manner (He et al., 2014). In addition, Zhou et al. showed that when the endogenous Mx1 was knocked down in PK-15 cells, the replication of CSFV would increase (Zhou et al., 2018). Further exploration of the antiviral mechanism of Mx1 revealed that Mx1 protein restrained the RdRp activity of the CSFV NS5B (Zhou et al., 2018). A recent study demonstrated that after CSFV-infected PK15 cells, not only porcine Mx1 had the effect of resisting CSFV, but murine Mx1 could also dampen the replication of CSFV (Chen et al., 2020). Further identifying the mechanism of murine Mx1 (R614E) mutants against CSFV, it found that Mx1 (R614E) mutants interacted with the nucleocapsid protein(C) of CSFV to reduce virus titer (Chen et al., 2020).

#### 3.2.2 TRIM

Tripartite motif-containing protein 56 (TRIM56) is a member of the TRIM family expressed constitutively in most organizations. After cells respectively infected with BVDV, hepatitis C virus (HCV), and vesicular stomatitis virus (VSV), overexpression of TRIM56 led to a dramatic decline in BVDV titer without affecting the other two viruses (Wang et al., 2011). They found that TRIM56 targeted the life cycle of viral RNA replication and specifically restricted BVDV infection by interacting with N^pro^ (Wang et al., 2011).

#### 3.2.3 IFITM

Recently, Li et al. found that overexpression of IFN-induced transmembrane (IFITM) in porcine alveolar macrophages (PAM) could significantly dampen CSFV infection. When IFITMs were knockdown, the titer of CSFV in cells increased observably, indicating that the IFITM proteins could restrain CSFV *in vitro.* In addition, the results demonstrated that IFITMs did not interfere with the binding of CSFV to cells but restricted CSFV from entering cells (Li et al., 2019).

#### 3.2.4 ISG15

IFN-stimulated gene 15 (ISG15) is one of the earliest broad-spectrum ISGs, could be covalently coupled to the target protein *via* the C-terminal motif (LRLRGG), and also could regulate viral replication in the form of unbound free cytokines (Han et al., 2018). Studies showed that when CSFV infected PK-15 cells, IRF1 interacted with the binding site of the 5’-terminal promoter region of ISG15 to up-regulated the expression of ISG15 and then curbed the replication of CSFV (Li et al., 2018). Recent studies *via* overexpression and knockdown of ISG15 *in vitro* corroborated the inhibitory effect of ISG15 on CSFV in PAM. In addition, treatment of PAM with exogenous IFN-α could induce the expression of ISG15, thereby reducing the titer of CSFV (Li et al., 2020). Further exploration of the antiviral mechanism of this process revealed that ISG15 not only restrained the replication of CSFV through ISGylation but also dampened autophagy *via* ISGylation of autophagy protein BECN1, to prevent the up-regulation of CSFV induced by autophagy, thus ultimately inhibiting the proliferation of CSFV (Li et al., 2020).

#### 3.2.5 PKR

RNA-dependent protein kinase (PKR) recognized viral dsRNA through conserved dsRNA binding motifs (dsRBMs) and phosphorylated the eukaryotic translation initiation factor (eIF2α) to inhibit viral translation (Rothenburg & Brennan, 2020). Recently, Gil et al. have shown that different biotypes of BVDV infected the cells, and the activation of PKR was quite distinct. That CP BVDV infection could induce the activation of PKR and NF-κB, as well as the suppression of translation. However, NCP BVDV infection dampened the expression of PKR caused by poliovirus infection and inhibited the activation of NF-κB mediated by dsRNA (Gil et al., 2006b). On the basis of the above results, it is speculated that the specific repression of PKR by NCP BVDV may connect with the PI establishment of NCP BVDV (Gil et al., 2006b).

#### 3.2.6 OASL

Oligoadenylate synthetases-like (OASL) protein belongs to the OAS family. This family synthesized 2’-5’ oligoadenylates by recognizing the dsRNA produced by the virus, which then activated the latent RNase L, leading to RNA cleavage in the virus and cellular, ultimately dampening the synthesis of proteins *via* a broad-spectrum manner (Kristiansen et al., 2011). Intriguingly, depending on the different stages and mechanisms of virus infection, OASL could either inhibit or promote the replication of the virus (Choi et al., 2015). Among the genus *Pestivirus*, studies have revealed that infection of CSFV both *in vivo* and *in vitro* could induce the expression of pOASL. Furthermore, pOASL interacted with pMDA5 in RNase L-independent way and increased the production of IFN by enhancing pMDA5-mediated antiviral signal transduction, finally restraining CSFV replication (Li et al., 2017a).

#### 3.2.7 Viperin

Some explorations have indicated that the C-terminal domain of viperin may be related to combat the family *Flaviviridae* infections (Seo et al., 2011). Recently, Gizzi et al. observed that viperin protein could produce 3’-deoxy-3’,4’-didehydro-CTP (ddhCTP) through the radical S-adenosyl-L-methionine (SAM)-dependent mechanism, and then ddhCTP was used as the replication chain terminator of multiple members of the family *Flaviviridae* to directly terminated the replication of the virus in advance, thereby exerting antiviral actions (Gizzi et al., 2018). In the PK-15 cells, the titer of CSFV was significantly curbed by stably overexpressing of viperin. Further studies demonstrated that viperin did not influence the binding, entry, and release of CSFV but could act on the replication and/or translation. In addition, the study found that viperin interacted with CSFV E2 protein in the cytoplasm, indicating that viperin may restrain CSFV through this interaction course (Li et al., 2017b). Nevertheless, another research indicated that viperin interacted with the NS5A of CSFV (Xu et al., 2020). Xu et al. constructed three truncated domains of the viperin to explore the exact region that interacted with the NS5A and confirmed that the N-terminal domain played a significant role in inhibiting the replication of CSFV (Xu et al., 2020).

## 4 Strategies for pestiviruses to circumvent interferons

Interestingly, viruses also antagonize the production of IFNs in various ways to cripple the host antiviral response (Garcia-Sastre, 2017). Studies have found that some pestiviruses could antagonize IFNs through their self-encoded protein (**Figure 3**), causing IFNs to maintain a tolerable low level *in vivo*, wherefore the pestiviruses would not be eliminated, eventually might lead to a state of PI in the animal.

In the premier studies on BVDV infection, researchers discovered an intriguing phenomenon. Infection of CP BVDV could observably induce the expression of IFNs *in vitro*, whereas NCP BVDV infection curbed the production of IFNs. When NCP BVDV infected cows in early pregnancy (prior 125 days), fetuses might be born normally and develop into PI cattle after birth, while having specific immunotolerant to the infected strain. Some researchers demonstrated that the expression of multiple genes in PI fetal tissues is down-regulated, such as IFN-I transcription regulator, antigen presentation, T cell markers, B cell activation, and so on (Meyers et al., 2007; Knapek et al., 2020). Among them, inhibiting the production of IFN is a significant reason for the persistent infection of PI animals. However, in recent years, it has been discovered that the signal conduction of IFNs was not completely suppressed (Nilson et al., 2020). When PI fetuses with acute infection, it is found that a few activated immune cells could transiently induce the secretion of IFN-γ. The IFN-γ would enter the circulatory system and cooperate with the innate immune response, resulting in a distinct reduction of PI fetal viremia but could not completely eradicate the virus (Smirnova et al., 2014; Hansen et al., 2015).

When exploring the mechanism and characteristics of BVDV infection, investigators discovered that both NCP BVDV and CP BVDV blocked the expression of IFN-independent antiviral protein Mx but did not inhibit the induction of IFN-dependent Mx. Strikingly, the specific inhibitory effect mentioned above had no impact on the antiviral activities of IFNs against other viruses in cells (Schweizer et al., 2006). CSFV not only antagonized self-induced IFNs but also could blocking-up IFNs induced by other viruses (Ruggli et al., 2005; Luo et al., 2009a). Though the pestiviruses infection did not affect the antiviral properties of IFNs, owing to pestiviruses dampened the induction of IFNs by themselves and other viruses, it creates a low IFNs environment, thus increasing the replication of other co-infected viruses (Alkheraif et al., 2017). A growing number of studies have shown that the proteins of pestiviruses, such as N^pro^ and E^rns^, played a vital role in antagonizing the production of IFNs. Combining with recent studies on pestiviruses and taking BVDV and CSFV as examples, we will review the counteract of pestiviruses to IFNs in detail in the following sections.

### 4.1 N^pro^ inhibits IFNs production

#### 4.1.1 N^pro^ inhibits IFN-I production

##### 4.1.1.1 N^pro^-mediated the proteasomal degradation of IRF3

N^pro^ is the first protein encoded by the genome of the genus *Pestivirus*, which has autoprotease activity and can catalyze itself to break from the polyprotein and become a mature protein. IRF3 is one of the key molecules in the IFN-I production pathway. Studies have found that the N^pro^ protein of pestiviruses can act on IRF3 in various ways, thereby restraining the production of IFN-I. Overexpression of exogenous CSFV N^pro^ could dampen the IFN-α/β induced by CSFV or other viruses, and the results confirmed that CSFV N^pro^ protein is an antagonist to IFN induction (Ruggli et al., 2005). Moreover, the previous research of our group found that N^pro^ of NCP BVDV isolated from clinical pigs could inhibit IFN-I induced by poly(I:C) as well (Tao et al., 2017). In earlier studies on this antagonistic mechanism, La Rocca et al. showed that CSFV N^pro^ exerted antagonistic on IFN-α/β by repressing IRF3 transcriptional (La Rocca et al., 2005). However, BVDV N^pro^ could antagonize IFNs by curbing IRF3-mediated immune response, indicating that BVDV and CSFV N^pro^ might have different functions in impeding IFN-I (Horscroft et al., 2005). Different from the results of La Rocca, Bauhofer et al. showed that CSFV N^pro^ neither did not affect the transcriptional activity of the IRF3 promoter and the stability of IRF3 mRNA, nor did it interfere with the phosphorylation level and the nuclear translocation of IRF3, but *via* directly or indirectly targeted IRF3 for proteasomal degradation (Bauhofer et al., 2007; Seago et al., 2007). Interestingly, BVDV N^pro^ inhibited the activation of IRF3 and targeted IRF3 for polyubiquitination, and then IRF3 was degraded by the proteasome. Furthermore, even if IRF3 was not degraded and successfully translocated to the nucleus, BVDV N^pro^ could dampen the binding of IRF3 to DNA in the nucleus (Hilton et al., 2006). Unlike BVDV, IRF3 was rapidly degraded after CSFV infection, wherefore there was no report on whether N^pro^ prevented IRF3 from binding to DNA (Bauhofer et al., 2007). Indeed, pestiviruses N^pro^-mediated proteasomal degradation of IRF3 did not rely on other proteins but directly interacted with the binding site of N^pro^ on IRF3 to form a soluble complex. This process demanded the participation of full-length IRF3 no matter whether IRF3 is activated (Gottipati et al., 2016). In conclusion, the proteasome degradation of IRF3 mediated by pestiviruses N^pro^ is one of the essential mechanisms for pestiviruses antagonizing IFN-I.

Additionally, NCP/CP BVDV N^pro^ could restrain the activation of IRF3 through multiple signaling pathways (TRIF, TBK1, MDA5, and RIG-I), and both of them blocked the binding of IRF3 to DNA, ultimately repressing the production of IFN-β which was induced by poly(I:C). However, NCP/CP BVDV N^pro^ could not prevent the activation of NF-κB by poly(I:C) (Hilton et al., 2006). Intriguingly, although the IFN-I induced by BVDV was suppressed by viral proteins, NCP BVDV and CP BVDV could still produce a certain amount of IFNs after infecting pDC, and the total IFNs generated by CP BVDV was observably higher than that of NCP BVDV (Lee et al., 2008; Reid et al., 2016; Song et al., 2022). When investigating the reasons for this phenomenon, researchers found that NCP BVDV infection did not cause the activation of NF-κB, while CP BVDV could induce NF-κB activation (Gil et al., 2006b; Maldonado et al., 2020). In addition, CP BVDV infection significantly activated NF-κB, IRF1, and IRF7 to promote IFN-β expression, and the action of IRF7 was greater than that of IRF1 (Maldonado et al., 2020). NF-κB and IRF3 are enhancer components for the induction of IFN-I. We speculated that CP BVDV infection induces IFN-I may be due to the following two reasons. On the one hand, CP BVDV infection results in the activation of NF-κB. Alternatively, there may be other regulatory factors that can replace IRF3 to a certain extent, like IRF1 or IRF7, wherefore CP BVDV infection can dramatically induce IFN-I. In terms of NCP BVDV, since it cannot activate NF-κB after infection, although there may be other regulatory factors that can substitute IRF3, it still cannot distinctly induce the production of IFN-I.

The half-life of CSFV N^pro^ protein in PK-15 cells is about 4 hours, and it is degraded by ubiquitin-dependent proteasomal degradation in the cytoplasm (Seago et al., 2010). Pestivirus N^pro^ is known to be an unstable autoprotease located at the N-terminus of the genome. In order to clarify whether the degradation of IRF3 by pestivirus N^pro^ was related to the autoprotease activity of N^pro^, the specific deletion mutants of two amino acids of CSFV N^pro^ (C 112 and D 136) were obtained, which did not affect the activity of N^pro^ autoprotease but lost the ability to degrade IRF3. The results indicated that the degradation of IRF3 by N^pro^ was independent of the proteolytic activity of N^pro^ (Ruggli et al., 2009). Analogously, BVDV N^pro^ targeting IRF3 degradation is also independent of autoprotease activity (Gil et al., 2006a). According to the results of previous researchers, the mutation of N^pro^ made it lose the ability to target IRF3 for degradation and then lose the inhibitory impact on IFN-I, leading to the phenomenon that the host induces high levels of IFN-I to restrain pestivirus replication. Unexpectedly, mutants (C112R and D136N) of CSFV N^pro^ lost the ability to degrade IRF3, but still retained the virulence *in vivo*, and there was no attenuation or only partial attenuation. To some extent, these results suggested that there may be other proteins in the pestivirus that could curb the expression of IFN-I (Ruggli et al., 2009). Further, *in vivo* research revealed the mutant amino acid site in the live attenuated vaccine had no recovery during the continuous passage, which derived from the CSFV N^pro^ mutant (D136N), and the induction of IFN-I was not dampened (Tamura et al., 2014). Intriguingly, if the mutant amino acid restores to the original amino acid (N136D), the degradation ability of CSFV N^pro^ to IRF3 would resume, and the property of inhibiting IFN-I would also recover. Simultaneously, the replication ability and pathogenicity of CSFV were also enhanced, indicating that N^pro^ played a significant role in the pathogenicity of CSFV in pigs (Tamura et al., 2014). In short, the autoprotease activity of pestivirus N^pro^ does not influence the degradation of IRF3 and is not necessary for pestivirus to circumvent the antiviral effect of IFN-I.

Moreover, N^pro^ is a metalloprotein. The crystal structure prediction of CSFV N^pro^ revealed that N^pro^ had a unique protease fold comprised of a cysteine protease domain and a zinc-binding domain (located at the C-terminal) (Gottipati et al., 2013). The zinc-binding domain contained a conserved metal-binding TRASH motif (Cys112-X21-Cys134-X3-Cys138, X represents any amino acid) (Szymanski et al., 2009; Gottipati et al., 2013). Szymanski et al. demonstrated that the cysteine residue in the TRASH motif played a vital role in the protein stability and zinc-binding of N^pro^. The deletion of the cysteine residue led to N^pro^ losing the ability to degrade IRF3 (Szymanski et al., 2009). In addition to the involvement of the TRASH motif in regulating the stability of CSFV N^pro^, the amino acid residues in the N-terminal domain of N^pro^ also played an essential role in the stability of N^pro^ and *via* regulation the interaction between N^pro^ and IRF3 to participate in regulating the production of IFN-I (Mine et al., 2015). Overall, the TRASH motif of pestivirus N^pro^ is essential for targeting the polyubiquitination degradation of IRF3, suggesting that the TRASH motif is crucial for pestivirus to restrain IFN.

##### 4.1.1.2 N^pro^ inhibits IFN-I expression in an IRF7-dependent manner

Indeed, apart from inhibiting the expression of IFN-I *via* interreaction with IRF3, CSFV N^pro^ also interacted with the cytokine IRF7 in pDC, and the interaction required the almost complete structure of IRF7 to partake. The TRASH motif restrained the expression of IFN-I in IRF7 dependent manner, but this course may not be crucial, and the concrete mechanism of the interplay between N^pro^ and IRF7 is still unclear (Fiebach et al., 2011).

##### 4.1.1.3 N^pro^ inhibits the expression of other interactant cellular proteins

As we all know, the successful infection of pathogenic microorganisms inevitably requires the involvement of host cells. S100A9, a member of the cellular danger-associated molecular patterns (DAMPs), promotes the activation of NF-κB through the TLR4/MyD88 pathway and then activates the expression of IFN-I. When pestivirus infection, BVDV N^pro^ interacted with the S100A9 to restrain IFN-I expression by decreasing the availability/activity of S100A9 in cells (Darweesh et al., 2018). Besides, CSFV N^pro^ specificity interplay with the ribosomal protein uS10 to reduce the production of uS10 in a proteasomal-dependent manner and inhibited the expression of TLR3 to facilitate CSFV replication (Lv et al., 2017b).

#### 4.1.2 N^pro^ inhibits IFN-III production

Recent studies suggested that CSFV N^pro^ not only dampened the production of IFN-I but also could restrain IFN-III by inhibiting the promoter activity and the expression of IRF1, as well as blocking the nuclear translocation of IRF1 (Cao et al., 2019a). Our study found that BVDV N^pro^ also inhibited IFN-III expression (unpublished data). These discoveries provide new insight for understanding the inhibition mechanisms of N^pro^ on IFNs and display more comprehensive mechanisms by which the virus escapes the host immune response. Overall, pestiviruses use N^pro^ protein to restrain IFNs of the host in diverse ways, which is one of the significant causes for pestiviruses establishing PI.

### 4.2 E^rns^ inhibits IFNs production

Furthermore, in addition to the suppression of IFN-I by N^pro^, E^rns^, a highly glycosylated protein, has continuously been pointed out that blocking the expression of IFN-I (de Martin & Schweizer, 2022). The envelope protein E^rns^ could interact with receptors on the cell surface and *via* energy-dependent clathrin-mediated endocytosis internalized into the cell to participate in the regulation of IFN-I (Zurcher et al., 2014a). Soluble BVDV E^rns^ could curb IFN-I induced by ssRNA and extracellular (rather than intracellular) dsRNA with its RNase activity (Magkouras et al., 2008; Matzener et al., 2009; Zurcher et al., 2014a; de Martin & Schweizer, 2022). The RNase activity of BVDV E^rns^ was essential for blocking IFN-I, and the C-terminus of BVDV E^rns^ also played a vital role in suppressing the IFN-I (Zurcher et al., 2014a). Analogously, the E^rns^ of CSFV restrained IFN-β induced by poly(I:C) in a dose-dependent manner, as well as blocking IFN-β mediated by other viral infections (Chen et al., 2007; Luo et al., 2009a). Unlike BVDV, the RNase activity of E^rns^ was not necessary for this inhibition course (Luo et al., 2009a). N-linked glycosylation of CSFV E^rns^ was requisite for suppressing poly(I:C)-induced IFN-β (Luo et al., 2009b), indicating that the E^rns^ of BVDV and CSFV restrains IFN through disparate mechanisms.

Human antimicrobial peptide LL37 forms a complex with RNA and acts as a protective agent for RNA. The study found that the complex was not degraded by the RNase of pestivirus E^rns^ *in vitro*, whereas it could be degraded by E^rns^ RNase intracellularly, and that the TLR3-dependent IFN synthesis activated by the complex was also restrained by pestivirus E^rns^ (Zurcher et al., 2014b). It revealed that pestivirus E^rns^ was an antagonist of IFN, and RNase activity played an important role in controlling IFN (Zurcher et al., 2014b). In addition to the RNase activity of E^rns^, it has also been speculated that E^rns^ inhibition of dsRNA depends on the homodimer form of E^rns^. But then some researchers showed that monomeric E^rns^ also could cleave dsRNA and the induction of IFN by dsRNA under *in vitro* conditions (Lussi et al., 2018). In brief, the antagonism of E^rns^ on IFNs is independent of its existing form.

Both N^pro^ and E^rns^ are antagonists of IFN. The puzzling thing is, are they redundant when establishing PI in the host? Or is there any interaction between the two? Although PI animals carry the virus for their whole life long, not all cells of PI animals are infected by the pestivirus. N^pro^ only exists in the infected cells, while the secreted E^rns^ can circulate throughout the body in the bloodstream and act on uninfected cells (Peterhans & Schweizer, 2013; Tautz et al., 2015). Indeed, N^pro^ and E^rns^ are not redundant and inhibit pestivirus-induced IFN-I at different levels (Schweizer & Peterhans, 2014; Tautz et al., 2015). N-terminal of BVDV N^pro^ lacking protease activity or E^rns^ of BVDV lacking RNase activity infected pregnant cows, both of them could lead to attenuation in the adult host but could not preclude the formation of the PI fetus. However, the double mutant of N^pro^ and E^rns^ above-mentioned could significantly induce the production of IFN-I, thereby preventing the establishment of the PI fetus, indicating that N^pro^ and E^rns^ have a synergistic effect in the formation of PI (Meyers et al., 2007). Furthermore, Tao et al. of our group discovered that N^pro^ His49 and E^rns^ Lys412 were the crux amino acid sites in this course (Tao et al., 2018). In short, BVDV N^pro^ and E^rns^ are non-redundant antagonists of IFN that cooperate to escape the host’s antiviral response *in vivo* and *in vitro*.

### 4.3 Other viral proteins inhibit IFN production

Apart from N^pro^ and E^rns^, there are also other proteins in pestivirus to curb IFNs. Early studies have shown that the protease activity of BVDV NS3 could not block the activation of the IFN-β promoter, indicating that NS3 has no inhibitory property on the transcriptional level of IFN-β (Gamlen et al., 2010). However, after CSFV infection, NS3 of CSFV could interact with tumor necrosis factor receptor-associated factor 6 (TRAF6). On the one hand, TRAF6 activated the NF-κB pathway and induced the expression of IFN-β and IL-6 to inhibit CSFV replication. On the other hand, CSFV NS3 promoted CSFV replication by degrading TRAF6 and antagonizing TRAF6-activated antiviral responses, suggesting that CSFV NS3 could also protect CSFV from host antiviral defenses (Lv et al., 2017a). CSFV NS4B could restrain the expression of IL-6, IL-8, and IFN-β to a certain extent by inhibiting MAVS-mediated IRF3 protein expression and NF-κB phosphorylation (Cao et al., 2019b; Dong et al., 2022). Moreover, BVDV NS4B protein could interact with the N-terminal caspase recruitment domain (CARD) of MDA5, inhibiting the MDA5-mediated signal transduction pathway to antagonize the production of IFN-β (Shan et al., 2021).

Organisms employ various mechanisms to fight viral infection, such as directly inducing the production of IFNs to affect virus replication, and regulating autophagy and apoptosis to influence the virus from infecting cells. Studies have found that RNA virus infection caused RLR-induced IRF3-mediated apoptosis was another effective way to reduce virus transmission. This apoptotic response was triggered by the specific lysine ubiquitination of IRF3, which was independent of the transcriptional activation mechanism of IRF3 (Chattopadhyay et al., 2016; Chattopadhyay & Sen, 2017). Indeed, IRF3 has dual functions in the course of the antiviral response. IRF3 degradation by CSFV N^pro^ not only could inhibit MAVS-mediated IFN-I expression but also blocked MAVS and IRF3-mediated mitochondrial apoptosis pathways (Gou et al., 2017; Dong et al., 2018; Hardy et al., 2020). Jefferson et al. demonstrated that pestivirus N^pro^ curbed endogenous mitochondrial apoptosis by inhibiting the activation of the IRF3-dependent pro-apoptotic protein Bcl2-Associated X (BAX), indicating that mitochondria and peroxisomes may be new sites for N^pro^ to regulate IRF3 (Jefferson et al., 2014). Additionally, pestivirus infection induced autophagy to promote virus replication (Pei et al., 2014; Oguejiofor et al., 2019; Zhang et al., 2021). Explored the mechanism and found that CSFV-mediated autophagy restrained the expression of IFN-I. This inhibition process was related to the interplay between MAVS and BECN1, and mitogen-activated protein kinase (MAPK/ERK) signaling worked an essential role (Xie et al., 2021). As can be seen from the foregoing description, N^pro^ can interact with multiple host proteins to antagonize the antiviral effects of the host through disparate mechanisms (**Table 1**). It is curious whether N^pro^ also involves in the regulation of autophagy. And what role does N^pro^ play? These questions may shed new light on an in-depth exploration of N^pro^ in the future.

**Table 1:** N^pro^ interacts with host proteins to antagonize the antiviral effects of the host through disparate mechanisms.

Interacting host protein	Antagonistic host antiviral methods	Mechanism	Reference
IRF3	Antagonism the production of IFN-I	Targeting IRF3 for proteasomal degradation	(Bauhofer et al., 2007; Seago et al., 2007)
IRF7	Antagonism the production of IFN-I	Unclear	(Fiebach et al., 2011)
S100A9	Antagonism the production of IFN-I	Reduce the availability/activity of S100A9 in cells	(Darweesh et al., 2018)
uS10	Antagonism the production of IFN-I	Suppress the production of uS10	(Lv et al., 2017b)
IRF1	Antagonism the production of IFN-III	Block the expression and nuclear translocation of IRF1	(Cao et al., 2019a)
BAX	Inhibit apoptosis	Restrain the activation of BAX	(Jefferson et al., 2014)

## 5 Conclusions and perspectives

Members of pestiviruses have a wide broad range of hosts and can cause cross-infection. Moreover, common pestiviruses (such as BVDV, CSFV, and BDV) can give rise to persistent infections of the host, causing them to carry and shed viruses for lifelong without inducing the production of antibodies. Therefore, this is also one of the critical reasons why pestiviruses are hard to eradicate and easy to pose a severe threat to economic development. Herein, we elaborate that pestiviruses infection can be recognized by a variety of PRRs in the host, thereby promoting the synthesis of IFNs, then inducing the secretion of multiple ISGs through the cascade of signal molecules, and finally exerting an antiviral effect. In addition, we also overview that pestiviruses can use their self-encoded proteins to antagonize the host antiviral response. In particular, the N^pro^ protein which is unique to pestivirus promotes immune escape by inhibiting the production of IFN, apoptosis, and other pathways. Thereinto, the repression of pestiviruses in IFNs has received extensive attention and in-depth research. Hereon, we reviewed the interaction between pestiviruses and IFNs, aiming to provide the theoretical underpinning for exploring the mechanism of immune escape by pestiviruses.

Studies on pestiviruses and IFNs are rich and diverse, and we apologize for the research not being covered in this article. Nevertheless, there is still a long way to go to explore their mutual regulation, and future research can focus on the following aspects: (I) We know that IFN-I and IFN-III are two non-redundant antiviral proteins that they are not only similar in signal transduction but also have many resemblances to their biological functions. At present, there are lots of studies on the interaction between pestiviruses and IFN-I, whereas the exploration of IFN-III is rare and still in the initial stage. Therefore, revealing the specific regulatory mechanism of pestiviruses infection on the IFN-III signaling pathway and clarifying the role of N^pro^ and E^rns^ proteins during the regulation can be future directions and sheds new light on researching the infection of pestiviruses. (II) In addition to the several pestiviruses proteins involved in IFNs regulation introduced in this overview, whether other proteins participated in the mutual regulation of pestiviruses and IFNs, and which signal molecules do they specifically act on in the IFN signaling pathway? These questions still require to be answered. (III) We already know that different types of IFNs have imparity prophylactic or therapeutic effects on different pestiviruses. However, at which stage of the pestivirus life cycle do different types of IFNs specifically act? Which ISGs are regulated by each kind of IFN to exert inhibitory properties, and what are the similarities and differences in their mechanisms, these issues remain equally unresolved and need to be further explored. In conclusion, the in-depth probe of the above problems will be helpful for the study of immune escape characteristics and pathogenesis mechanism of pestiviruses, to provide new ideas and means for the prevention and control of pestiviruses.

## Author contributions

TH designed and wrote the manuscript. GZ, CZ, YY, and PW critically read and corrected the manuscript. All authors contributed to the article and approved the submitted version.

## References

[B1] Alkheraif A. A. Topliff C. L. Reddy J. Massilamany C. Donis R. O. Meyers G.. (2017). Type 2 BVDV n(pro) suppresses IFN-1 pathway signaling in bovine cells and augments BRSV replication. Virology 507, 123–134. doi: 10.1016/j.virol.2017.04.015 28432927

[B2] Bauhofer O. Summerfield A. Sakoda Y. Tratschin J. D. Hofmann M. A. Ruggli N. (2007). Classical swine fever virus npro interacts with interferon regulatory factor 3 and induces its proteasomal degradation. J. Virol. 81 (7), 3087–3096. doi: 10.1128/JVI.02032-06 17215286 PMC1866024

[B3] Cai B. Bai Q. Chi X. Goraya M. U. Wang L. Wang S.. (2017). Infection with classical swine fever virus induces expression of type III interferons and activates innate immune signaling. Front. Microbiol. 8, 2558. doi: 10.3389/fmicb.2017.02558 29312239 PMC5742159

[B4] Cao Z. Guo K. Zheng M. Ning P. Li H. Kang K.. (2015). A comparison of the impact of shimen and c strains of classical swine fever virus on toll-like receptor expression. J. Gen. Virol. 96 (Pt 7), 1732–1745. doi: 10.1099/vir.0.000129 25805409

[B5] Cao T. Li X. Xu Y. Zhang S. Wang Z. Shan Y.. (2019a). Npro of classical swine fever virus suppresses type III interferon production by inhibiting IRF1 expression and its nuclear translocation. Viruses 11 (11), 998. doi: 10.3390/v11110998 31683525 PMC6893713

[B6] Cao Z. Yang Q. Zheng M. Lv H. Kang K. Zhang Y. (2019b). Classical swine fever virus non-structural proteins modulate toll-like receptor signaling pathways in porcine monocyte-derived macrophages. Vet. Microbiol. 230, 101–109. doi: 10.1016/j.vetmic.2019.01.025 30827374

[B7] Chattopadhyay S. Kuzmanovic T. Zhang Y. Wetzel J. L. Sen G. C. (2016). Ubiquitination of the transcription factor IRF-3 activates RIPA, the apoptotic pathway that protects mice from viral pathogenesis. Immunity 44 (5), 1151–1161. doi: 10.1016/j.immuni.2016.04.009 27178468 PMC4991351

[B8] Chattopadhyay S. Sen G. C. (2017). RIG-i-like receptor-induced IRF3 mediated pathway of apoptosis (RIPA): a new antiviral pathway. Protein Cell 8 (3), 165–168. doi: 10.1007/s13238-016-0334-x 27815826 PMC5326620

[B9] Chen J. Wu Y. Wu X. D. Zhou J. Liang X. D. Baloch A. S.. (2020). The R614E mutation of mouse Mx1 protein contributes to the novel antiviral activity against classical swine fever virus. Vet. Microbiol. 243, 108621. doi: 10.1016/j.vetmic.2020.108621 32273007

[B10] Chen L. Xia Y. H. Pan Z. S. Zhang C. Y. (2007). Expression and functional characterization of classical swine fever virus e(rns) protein. Protein Expr Purif 55 (2), 379–387. doi: 10.1016/j.pep.2007.05.003 17587595

[B11] Choi U. Y. Kang J. S. Hwang Y. S. Kim Y. J. (2015). Oligoadenylate synthase-like (OASL) proteins: Dual functions and associations with diseases. Exp. Mol. Med. 47, e144. doi: 10.1038/emm.2014.110 25744296 PMC4351405

[B12] Darweesh M. F. Rajput M. K. S. Braun L. J. Rohila J. S. Chase C. C. L. (2018). BVDV npro protein mediates the BVDV induced immunosuppression through interaction with cellular S100A9 protein. Microb. Pathog. 121, 341–349. doi: 10.1016/j.micpath.2018.05.047 29859294 PMC7127600

[B13] de Martin E. Schweizer M. (2022). Fifty shades of e(rns): innate immune evasion by the viral endonucleases of all pestivirus species. Viruses 14 (2), 265. doi: 10.3390/v14020265 35215858 PMC8880635

[B14] Deng M. Chen N. Guidarini C. Xu Z. Zhang J. Cai L.. (2020). Prevalence and genetic diversity of bovine viral diarrhea virus in dairy herds of China. Vet. Microbiol. 242, 108565. doi: 10.1016/j.vetmic.2019.108565 32122580

[B15] Dong W. Jing H. Wang H. Cao S. Sun Y. Zhang Y.. (2022). Classical swine fever virus NS4B protein interacts with MAVS and inhibits IL-8 expression in PAMs. Virus Res. 307, 198622. doi: 10.1016/j.virusres.2021.198622 34762991

[B16] Dong X. Y. Liu W. J. Zhao M. Q. Wang J. Y. Pei J. J. Luo Y. W.. (2013). Classical swine fever virus triggers RIG-I and MDA5-dependent signaling pathway to IRF-3 and NF-κB activation to promote secretion of interferon and inflammatory cytokines in porcine alveolar macrophages. Virol. J. 10, 286. doi: 10.1186/1743-422X-10-286 24034559 PMC3849481

[B17] Dong W. Lv H. Li C. Liu Y. Wang C. Lin J.. (2018). MAVS induces a host cell defense to inhibit CSFV infection. Arch. Virol. 163 (7), 1805–1821. doi: 10.1007/s00705-018-3804-z 29556776

[B18] Elsheikh A. A. Braun L. J. Mansour S. M. G. Orabi A. Alqahtani A. S. Benfield D. A.. (2019). The effect of human interferon alpha on replication of different bovine viral diarrhea virus strains. Acta Virol. 63 (3), 261–269. doi: 10.4149/av_2019_303 31507191

[B19] Fan S. Wu K. Zhao M. Yuan J. Ma S. Zhu E.. (2021). LDHB inhibition induces mitophagy and facilitates the progression of CSFV infection. Autophagy 17 (9), 2305–2324. doi: 10.1080/15548627.2020.1823123 32924761 PMC8496725

[B20] Fensterl V. Chattopadhyay S. Sen G. C. (2015). No love lost between viruses and interferons. Annu. Rev. Virol. 2 (1), 549–572. doi: 10.1146/annurev-virology-100114-055249 26958928 PMC9549753

[B21] Fiebach A. R. Guzylack-Piriou L. Python S. Summerfield A. Ruggli N. (2011). Classical swine fever virus n(pro) limits type I interferon induction in plasmacytoid dendritic cells by interacting with interferon regulatory factor 7. J. Virol. 85 (16), 8002–8011. doi: 10.1128/JVI.00330-11 21680532 PMC3147952

[B22] Forero A. Ozarkar S. Li H. Lee C. H. Hemann E. A. Nadjsombati M. S.. (2019). Differential activation of the transcription factor IRF1 underlies the distinct immune responses elicited by type I and type III interferons. Immunity 51 (3), 451–464 e6. doi: 10.1016/j.immuni.2019.07.007 31471108 PMC7447158

[B23] Gamlen T. Richards K. H. Mankouri J. Hudson L. McCauley J. Harris M.. (2010). Expression of the NS3 protease of cytopathogenic bovine viral diarrhea virus results in the induction of apoptosis but does not block activation of the beta interferon promoter. J. Gen. Virol. 91 (Pt 1), 133–144. doi: 10.1099/vir.0.016170-0 19793904

[B24] Garcia-Sastre A. (2017). Ten strategies of interferon evasion by viruses. Cell Host Microbe 22 (2), 176–184. doi: 10.1016/j.chom.2017.07.012 28799903 PMC5576560

[B25] Gil L. H. Ansari I. H. Vassilev V. Liang D. Lai V. C. Zhong W.. (2006a). The amino-terminal domain of bovine viral diarrhea virus npro protein is necessary for alpha/beta interferon antagonism. J. Virol. 80 (2), 900–911. doi: 10.1128/JVI.80.2.900-911.2006 16378992 PMC1346884

[B26] Gil L. H. van Olphen A. L. Mittal S. K. Donis R. O. (2006b). Modulation of PKR activity in cells infected by bovine viral diarrhea virus. Virus Res. 116 (1-2), 69–77. doi: 10.1016/j.virusres.2005.08.011 16194578

[B27] Gillespie J. H. Coggins L. Thompson J. Baker J. A. (1961). Comparison by neutralization tests of strains of virus isolated from virus diarrhea and mucosal disease. Cornell Vet. 51, 155–159. 13705336

[B28] Gizzi A. S. Grove T. L. Arnold J. J. Jose J. Jangra R. K. Garforth S. J.. (2018). A naturally occurring antiviral ribonucleotide encoded by the human genome. Nature 558 (7711), 610–614. doi: 10.1038/s41586-018-0238-4 29925952 PMC6026066

[B29] Gottipati K. Holthauzen L. M. Ruggli N. Choi K. H. (2016). Pestivirus npro directly interacts with interferon regulatory factor 3 monomer and dimer. J. Virol. 90 (17), 7740–7747. doi: 10.1128/JVI.00318-16 27334592 PMC4988160

[B30] Gottipati K. Ruggli N. Gerber M. Tratschin J. D. Benning M. Bellamy H.. (2013). The structure of classical swine fever virus n(pro): a novel cysteine autoprotease and zinc-binding protein involved in subversion of type I interferon induction. PloS Pathog. 9 (10), e1003704. doi: 10.1371/journal.ppat.1003704 24146623 PMC3798407

[B31] Gou H. Zhao M. Xu H. Yuan J. He W. Zhu M.. (2017). CSFV induced mitochondrial fission and mitophagy to inhibit apoptosis. Oncotarget 8 (24), 39382–39400. doi: 10.18632/oncotarget.17030 28455958 PMC5503620

[B32] Guo Y. Song Z. Cheng X. Wang Y. Luo X. An R.. (2020). Molecular and functional characterization of ovis aries IFN-epsilon. Mol. Immunol. 119, 1–7. doi: 10.1016/j.molimm.2020.01.001 31926433

[B33] Haller O. Kochs G. (2011). Human MxA protein: an interferon-induced dynamin-like GTPase with broad antiviral activity. J. Interferon Cytokine Res. 31 (1), 79–87. doi: 10.1089/jir.2010.0076 21166595

[B34] Han H. G. Moon H. W. Jeon Y. J. (2018). ISG15 in cancer: Beyond ubiquitin-like protein. Cancer Lett. 438, 52–62. doi: 10.1016/j.canlet.2018.09.007 30213559

[B35] Hansen T. R. Smirnova N. P. Webb B. T. Bielefeldt-Ohmann H. Sacco R. E. Van Campen H. (2015). Innate and adaptive immune responses to *in utero* infection with bovine viral diarrhea virus. Anim. Health Res. Rev. 16 (1), 15–26. doi: 10.1017/S1466252315000122 26050568

[B36] Hardy S. Jackson B. Goodbourn S. Seago J. (2020). Classical swine fever virus n(pro) antagonises IRF3 to prevent IFN-independent TLR3 and RIG-i-mediated apoptosis. J. Virol. 95 (5), e01136–e01120. doi: 10.1128/JVI.01136-20 33328306 PMC8092839

[B37] He D. N. Zhang X. M. Liu K. Pang R. Zhao J. Zhou B.. (2014). *In vitro* inhibition of the replication of classical swine fever virus by porcine Mx1 protein. Antiviral Res. 104, 128–135. doi: 10.1016/j.antiviral.2014.01.020 24500530

[B38] Hilton L. Moganeradj K. Zhang G. Chen Y. H. Randall R. E. McCauley J. W.. (2006). The NPro product of bovine viral diarrhea virus inhibits DNA binding by interferon regulatory factor 3 and targets it for proteasomal degradation. J. Virol. 80 (23), 11723–11732. doi: 10.1128/JVI.01145-06 16971436 PMC1642611

[B39] Holzinger D. Jorns C. Stertz S. Boisson-Dupuis S. Thimme R. Weidmann M.. (2007). Induction of MxA gene expression by influenza a virus requires type I or type III interferon signaling. J. Virol. 81 (14), 7776–7785. doi: 10.1128/JVI.00546-06 17494065 PMC1933351

[B40] Horscroft N. Bellows D. Ansari I. Lai V. C. Dempsey S. Liang D.. (2005). Establishment of a subgenomic replicon for bovine viral diarrhea virus in huh-7 cells and modulation of interferon-regulated factor 3-mediated antiviral response. J. Virol. 79 (5), 2788–2796. doi: 10.1128/JVI.79.5.2788-2796.2005 15708997 PMC548457

[B41] Husser L. Alves M. P. Ruggli N. Summerfield A. (2011). Identification of the role of RIG-I, MDA-5 and TLR3 in sensing RNA viruses in porcine epithelial cells using lentivirus-driven RNA interference. Virus Res. 159 (1), 9–16. doi: 10.1016/j.virusres.2011.04.005 21539869

[B42] Jefferson M. Whelband M. Mohorianu I. Powell P. P. (2014). The pestivirus n terminal protease n(pro) redistributes to mitochondria and peroxisomes suggesting new sites for regulation of IRF3 by n(pro.). PloS One 9 (2), e88838. doi: 10.1371/journal.pone.0088838 24551175 PMC3925175

[B43] Jo W. K. van Elk C. van de Bildt M. van Run P. Petry M. Jesse S. T.. (2019). An evolutionary divergent pestivirus lacking the n(pro) gene systemically infects a whale species. Emerg. Microbes Infect. 8 (1), 1383–1392. doi: 10.1080/22221751.2019.1664940 31526243 PMC6758615

[B44] Johnston C. M. Fahnoe U. Lohse L. Bukh J. Belsham G. J. Rasmussen T. B. (2020). Analysis of virus population profiles within pigs infected with virulent classical swine fever viruses; evidence for bottlenecks in transmission but absence of tissue specific virus variants. J. Virol. 94 (19), e01119–e01120. doi: 10.1128/JVI.01119-20 32699086 PMC7495388

[B45] Klimowicz-Bodys M. D. Polak M. P. Ploneczka-Janeczko K. Bagnicka E. Zbroja D. Rypula K. (2022). Lack of fetal protection against bovine viral diarrhea virus in a vaccinated heifer. Viruses 14 (2), 311. doi: 10.3390/v14020311 35215904 PMC8879756

[B46] Knapek K. J. Georges H. M. Van Campen H. Bishop J. V. Bielefeldt-Ohmann H. Smirnova N. P.. (2020). Fetal lymphoid organ immune responses to transient and persistent infection with bovine viral diarrhea virus. Viruses 12 (8), 816. doi: 10.3390/v12080816 32731575 PMC7472107

[B47] Kristiansen H. Gad H. H. Eskildsen-Larsen S. Despres P. Hartmann R. (2011). The oligoadenylate synthetase family: an ancient protein family with multiple antiviral activities. J. Interferon Cytokine Res. 31 (1), 41–47. doi: 10.1089/jir.2010.0107 21142819

[B48] La Rocca S. A. Herbert R. J. Crooke H. Drew T. W. Wileman T. E. Powell P. P. (2005). Loss of interferon regulatory factor 3 in cells infected with classical swine fever virus involves the n-terminal protease, npro. J. Virol. 79 (11), 7239–7247. doi: 10.1128/JVI.79.11.7239-7247.2005 15890962 PMC1112113

[B49] Lee S. R. Pharr G. T. Boyd B. L. Pinchuk L. M. (2008). Bovine viral diarrhea viruses modulate toll-like receptors, cytokines and co-stimulatory molecules genes expression in bovine peripheral blood monocytes. Comp. Immunol. Microbiol. Infect. Dis. 31 (5), 403–418. doi: 10.1016/j.cimid.2007.06.006 17706777

[B50] Li D. Dong H. Li S. Munir M. Chen J. Luo Y.. (2013). Hemoglobin subunit beta interacts with the capsid protein and antagonizes the growth of classical swine fever virus. J. Virol. 87 (10), 5707–5717. doi: 10.1128/JVI.03130-12 23487454 PMC3648164

[B51] Li X. Q. Li X. N. Liang J. J. Cai X. B. Tao Q. Li Y. X.. (2018). IRF1 up-regulates isg15 gene expression in dsRNA stimulation or CSFV infection by targeting nucleotides -487 to -325 in the 5' flanking region. Mol. Immunol. 94, 153–165. doi: 10.1016/j.molimm.2017.12.025 29324236

[B52] Li W. Mao L. Cao Y. Zhou B. Yang L. Han L.. (2017b). Porcine viperin protein inhibits the replication of classical swine fever virus (CSFV) *in vitro* . Virol. J. 14 (1), 202. doi: 10.1186/s12985-017-0868-4 29061156 PMC5654138

[B53] Li C. Wang Y. Zheng H. Dong W. Lv H. Lin J.. (2020). Antiviral activity of ISG15 against classical swine fever virus replication in porcine alveolar macrophages *via* inhibition of autophagy by ISGylating BECN1. Vet. Res. 51 (1), 22. doi: 10.1186/s13567-020-00753-5 32093773 PMC7038623

[B54] Li X. Xia Q. Meng C. Wu H. Huang H. Qian J.. (2021). Downregulation of SOCS gene expression can inhibit the formation of acute and persistent BDV infections. Scand. J. Immunol. 93 (1), e12974. doi: 10.1111/sji.12974 32910495

[B55] Li L. F. Yu J. Zhang Y. Yang Q. Li Y. Zhang L.. (2017a). Interferon-inducible oligoadenylate synthetase-like protein acts as an antiviral effector against classical swine fever virus *via* the MDA5-mediated type I interferon-signaling pathway. J. Virol. 91 (11), e01514–e01516. doi: 10.1128/JVI.01514-16 28331099 PMC5432864

[B56] Li C. Zheng H. Wang Y. Dong W. Liu Y. Zhang L.. (2019). Antiviral role of IFITM proteins in classical swine fever virus infection. Viruses 11 (2), 126. doi: 10.3390/v11020126 30704088 PMC6409519

[B57] Luo X. Ling D. Li T. Wan C. Zhang C. Pan Z. (2009a). Classical swine fever virus erns glycoprotein antagonizes induction of interferon-beta by double-stranded RNA. Can. J. Microbiol. 55 (6), 698–704. doi: 10.1139/w09-013 19767841

[B58] Luo X. Pan R. Wan C. Liu X. Wu J. Pan Z. (2009b). Glycosylation of classical swine fever virus e(rns) is essential for binding double-stranded RNA and preventing interferon-beta induction. Virus Res. 146 (1-2), 135–139. doi: 10.1016/j.virusres.2009.09.011 19782108

[B59] Lussi C. Sauter K. S. Schweizer M. (2018). Homodimerisation-independent cleavage of dsRNA by a pestiviral nicking endoribonuclease. Sci. Rep. 8 (1), 8226. doi: 10.1038/s41598-018-26557-4 29844335 PMC5974291

[B60] Lv H. Dong W. Cao Z. Li X. Wang J. Qian G.. (2017a). TRAF6 is a novel NS3-interacting protein that inhibits classical swine fever virus replication. Sci. Rep. 7 (1), 6737. doi: 10.1038/s41598-017-06934-1 28751780 PMC5532216

[B61] Lv H. Dong W. Qian G. Wang J. Li X. Cao Z.. (2017b). uS10, a novel npro-interacting protein, inhibits classical swine fever virus replication. J. Gen. Virol. 98 (7), 1679–1692. doi: 10.1099/jgv.0.000867 28721853

[B62] Ma Y. Wang L. Jiang X. Yao X. Huang X. Zhou K.. (2022). Integrative transcriptomics and proteomics analysis provide a deep insight into bovine viral diarrhea virus-host interactions during BVDV infection. Front. Immunol. 13, 862828. doi: 10.3389/fimmu.2022.862828 35371109 PMC8966686

[B63] Magkouras I. Matzener P. Rumenapf T. Peterhans E. Schweizer M. (2008). RNase-dependent inhibition of extracellular, but not intracellular, dsRNA-induced interferon synthesis by erns of pestiviruses. J. Gen. Virol. 89 (Pt 10), 2501–2506. doi: 10.1099/vir.0.2008/003749-0 18796719

[B64] Maldonado N. Fredericksen F. Espineira C. Toledo C. Oltra J. la Barra V.. (2020). BVDV-1 induces interferon-beta gene expression through a pathway involving IRF1, IRF7, and NF-kappaB activation. Mol. Immunol. 128, 33–40. doi: 10.1016/j.molimm.2020.09.018 33053462

[B65] Matzener P. Magkouras I. Rumenapf T. Peterhans E. Schweizer M. (2009). The viral RNase e(rns) prevents IFN type-I triggering by pestiviral single- and double-stranded RNAs. Virus Res. 140 (1-2), 15–23. doi: 10.1016/j.virusres.2008.10.015 19041350

[B66] Meyers G. Ege A. Fetzer C. von Freyburg M. Elbers K. Carr V.. (2007). Bovine viral diarrhea virus: prevention of persistent fetal infection by a combination of two mutations affecting erns RNase and npro protease. J. Virol. 81 (7), 3327–3338. doi: 10.1128/JVI.02372-06 17215285 PMC1866084

[B67] Mine J. Tamura T. Mitsuhashi K. Okamatsu M. Parchariyanon S. Pinyochon W.. (2015). The n-terminal domain of npro of classical swine fever virus determines its stability and regulates type I IFN production. J. Gen. Virol. 96 (Pt 7), 1746–1756. doi: 10.1099/vir.0.000132 25809915

[B68] Mordstein M. Neugebauer E. Ditt V. Jessen B. Rieger T. Falcone V.. (2010). Lambda interferon renders epithelial cells of the respiratory and gastrointestinal tracts resistant to viral infections. J. Virol. 84 (11), 5670–5677. doi: 10.1128/JVI.00272-10 20335250 PMC2876583

[B69] Neill J. D. Workman A. M. Hesse R. Bai J. Porter E. P. Meadors B.. (2019). Identification of BVDV2b and 2c subgenotypes in the united states: Genetic and antigenic characterization. Virology 528, 19–29. doi: 10.1016/j.virol.2018.12.002 30553108

[B70] Nilson S. M. Workman A. M. Sjeklocha D. Brodersen B. Grotelueschen D. M. Petersen J. L. (2020). Upregulation of the type I interferon pathway in feedlot cattle persistently infected with bovine viral diarrhea virus. Virus Res. 278, 197862. doi: 10.1016/j.virusres.2020.197862 31926963

[B71] Oguejiofor C. F. Thomas C. Cheng Z. Wathes D. C. (2019). Mechanisms linking bovine viral diarrhea virus (BVDV) infection with infertility in cattle. Anim. Health Res. Rev. 20 (1), 72–85. doi: 10.1017/S1466252319000057 31895016

[B72] Onoguchi K. Yoneyama M. Takemura A. Akira S. Taniguchi T. Namiki H.. (2007). Viral infections activate types I and III interferon genes through a common mechanism. J. Biol. Chem. 282 (10), 7576–7581. doi: 10.1074/jbc.M608618200 17204473

[B73] Pei J. Zhao M. Ye Z. Gou H. Wang J. Yi L.. (2014). Autophagy enhances the replication of classical swine fever virus *in vitro* . Autophagy 10 (1), 93–110. doi: 10.4161/auto.26843 24262968 PMC4389882

[B74] Peterhans E. Schweizer M. (2010). Pestiviruses: How to outmaneuver your hosts. Vet. Microbiol. 142 (1-2), 18–25. doi: 10.1016/j.vetmic.2009.09.038 19846261

[B75] Peterhans E. Schweizer M. (2013). BVDV: a pestivirus inducing tolerance of the innate immune response. Biologicals 41 (1), 39–51. doi: 10.1016/j.biologicals.2012.07.006 22871358

[B76] Quintana M. E. Barone L. Forlenza M. B. Trotta M. V. Turco C. Mansilla F. C.. (2018). A direct high-throughput in cell-ELISA for measuring infectivity of cytopathic and non-cytopathic bovine viral diarrhoea virus strains applied to the assessment of antiviral activity. J. Virol. Methods 260, 75–81. doi: 10.1016/j.jviromet.2018.07.010 30031751

[B77] Quintana M. E. Barone L. J. Trotta M. V. Turco C. Mansilla F. C. Capozzo A. V.. (2020a). In-vivo activity of IFN-lambda and IFN-alpha against bovine-viral-diarrhea virus in a mouse model. Front. Vet. Sci. 7, 45. doi: 10.3389/fvets.2020.00045 32118067 PMC7015039

[B78] Quintana M. E. Cardoso N. P. Pereyra R. Barone L. J. Barrionuevo F. M. Mansilla F. C.. (2020b). Interferon lambda protects cattle against bovine viral diarrhea virus infection. Vet. Immunol. Immunopathol. 230, 110145. doi: 10.1016/j.vetimm.2020.110145 33160262

[B79] Raftery N. Stevenson N. J. (2017). Advances in anti-viral immune defence: revealing the importance of the IFN JAK/STAT pathway. Cell Mol. Life Sci. 74 (14), 2525–2535. doi: 10.1007/s00018-017-2520-2 28432378 PMC7079803

[B80] Reid E. Juleff N. Windsor M. Gubbins S. Roberts L. Morgan S.. (2016). Type I and III IFNs produced by plasmacytoid dendritic cells in response to a member of the flaviviridae suppress cellular immune responses. J. Immunol. 196 (10), 4214–4226. doi: 10.4049/jimmunol.1600049 27053760

[B81] Rothenburg S. Brennan G. (2020). Species-specific host-virus interactions: Implications for viral host range and virulence. Trends Microbiol. 28 (1), 46–56. doi: 10.1016/j.tim.2019.08.007 31597598 PMC6925338

[B82] Ruggli N. Bird B. H. Liu L. Bauhofer O. Tratschin J. D. Hofmann M. A. (2005). N(pro) of classical swine fever virus is an antagonist of double-stranded RNA-mediated apoptosis and IFN-alpha/beta induction. Virology 340 (2), 265–276. doi: 10.1016/j.virol.2005.06.033 16043207

[B83] Ruggli N. Summerfield A. Fiebach A. R. Guzylack-Piriou L. Bauhofer O. Lamm C. G.. (2009). Classical swine fever virus can remain virulent after specific elimination of the interferon regulatory factor 3-degrading function of npro. J. Virol. 83 (2), 817–829. doi: 10.1128/JVI.01509-08 18987150 PMC2612357

[B84] Schneider W. M. Chevillotte M. D. Rice C. M. (2014). Interferon-stimulated genes: a complex web of host defenses. Annu. Rev. Immunol. 32, 513–545. doi: 10.1146/annurev-immunol-032713-120231 24555472 PMC4313732

[B85] Schweizer M. Matzener P. Pfaffen G. Stalder H. Peterhans E. (2006). "Self" and "nonself" manipulation of interferon defense during persistent infection: Bovine viral diarrhea virus resists alpha/beta interferon without blocking antiviral activity against unrelated viruses replicating in its host cells. J. Virol. 80 (14), 6926–6935. doi: 10.1128/JVI.02443-05 16809298 PMC1489018

[B86] Schweizer M. Peterhans E. (2014). Pestiviruses. Annu. Rev. Anim. Biosci. 2, 141–163. doi: 10.1146/annurev-animal-022513-114209 25384138

[B87] Seago J. Goodbourn S. Charleston B. (2010). The classical swine fever virus npro product is degraded by cellular proteasomes in a manner that does not require interaction with interferon regulatory factor 3. J. Gen. Virol. 91 (Pt 3), 721–726. doi: 10.1099/vir.0.015545-0 19906943

[B88] Seago J. Hilton L. Reid E. Doceul V. Jeyatheesan J. Moganeradj K.. (2007). The npro product of classical swine fever virus and bovine viral diarrhea virus uses a conserved mechanism to target interferon regulatory factor-3. J. Gen. Virol. 88 (Pt 11), 3002–3006. doi: 10.1099/vir.0.82934-0 17947522

[B89] Segredo-Otero E. Sanjuan R. (2020). The role of spatial structure in the evolution of viral innate immunity evasion: A diffusion-reaction cellular automaton model. PloS Comput. Biol. 16 (2), e1007656. doi: 10.1371/journal.pcbi.1007656 32040504 PMC7034925

[B90] Seo J. Y. Yaneva R. Cresswell P. (2011). Viperin: A multifunctional, interferon-inducible protein that regulates virus replication. Cell Host Microbe 10 (6), 534–539. doi: 10.1016/j.chom.2011.11.004 22177558 PMC3246677

[B91] Shan Y. Tong Z. Jinzhu M. Yu L. Zecai Z. Chenhua W.. (2021). Bovine viral diarrhea virus NS4B protein interacts with 2CARD of MDA5 domain and negatively regulates the RLR-mediated IFN-beta production. Virus Res. 302, 198471. doi: 10.1016/j.virusres.2021.198471 34097933

[B92] Simmonds P. Becher P. Bukh J. Gould E. A. Meyers G. Monath T.. (2017). ICTV virus taxonomy profile: flaviviridae. J. Gen. Virol. 98 (1), 2–3. doi: 10.1099/jgv.0.000672 28218572 PMC5370391

[B93] Smirnova N. P. Webb B. T. McGill J. L. Schaut R. G. Bielefeldt-Ohmann H. Van Campen H.. (2014). Induction of interferon-gamma and downstream pathways during establishment of fetal persistent infection with bovine viral diarrhea virus. Virus Res. 183, 95–106. doi: 10.1016/j.virusres.2014.02.002 24530541

[B94] Smith D. B. Meyers G. Bukh J. Gould E. A. Monath T. Scott Muerhoff A.. (2017). Proposed revision to the taxonomy of the genus pestivirus, family flaviviridae. J. Gen. Virol. 98 (8), 2106–2112. doi: 10.1099/jgv.0.000873 28786787 PMC5656787

[B95] Sommereyns C. Paul S. Staeheli P. Michiels T. (2008). IFN-lambda (IFN-lambda) is expressed in a tissue-dependent fashion and primarily acts on epithelial cells *in vivo* . PloS Pathog. 4 (3), e1000017. doi: 10.1371/journal.ppat.1000017 18369468 PMC2265414

[B96] Song Q. Zhao X. Cao C. Duan M. Shao C. Jiang S.. (2022). Research advances on interferon (IFN) response during BVDV infection. Res. Vet. Sci. 149, 151–158. doi: 10.1016/j.rvsc.2022.04.011 35839708

[B97] Sozzi E. Righi C. Boldini M. Bazzucchi M. Pezzoni G. Gradassi M.. (2020). Cross-reactivity antibody response after vaccination with modified live and killed bovine viral diarrhoea virus (BVD) vaccines. Vaccines (Basel) 8 (3), 374. doi: 10.3390/vaccines8030374 32664468 PMC7565157

[B98] Szymanski M. R. Fiebach A. R. Tratschin J. D. Gut M. Ramanujam V. M. Gottipati K.. (2009). Zinc binding in pestivirus n(pro) is required for interferon regulatory factor 3 interaction and degradation. J. Mol. Biol. 391 (2), 438–449. doi: 10.1016/j.jmb.2009.06.040 19540847

[B99] Tamura T. Nagashima N. Ruggli N. Summerfield A. Kida H. Sakoda Y. (2014). Npro of classical swine fever virus contributes to pathogenicity in pigs by preventing type I interferon induction at local replication sites. Vet. Res. 45 (1), 47. doi: 10.1186/1297-9716-45-47 24742209 PMC4018971

[B100] Tao J. Li B. Chen J. Zhang C. Ma Y. Zhu G.. (2018). N(pro) His49 and e(rns) Lys412 mutations in pig bovine viral diarrhea virus type 2 synergistically enhance the cellular antiviral response. Virus Genes 54 (1), 57–66. doi: 10.1007/s11262-017-1506-3 28852929

[B101] Tao J. Liao J. Wang J. Zhang X. Zhang Q. Zhu L.. (2017). Pig BVDV-2 non-structural protein (N(pro)) links to cellular antiviral response *in vitro* . Virus Genes 53 (2), 233–239. doi: 10.1007/s11262-016-1410-2 27866318

[B102] Tautz N. Tews B. A. Meyers G. (2015). The molecular biology of pestiviruses. Adv. Virus Res. 93, 47–160. doi: 10.1016/bs.aivir.2015.03.002 26111586

[B103] Vilcek S. Nettleton P. F. (2006). Pestiviruses in wild animals. Vet. Microbiol. 116 (1-3), 1–12. doi: doi: 10.1016/j.vetmic.2006.06.003 16839713

[B104] Walker F. C. Sridhar P. R. Baldridge M. T. (2021). Differential roles of interferons in innate responses to mucosal viral infections. Trends Immunol. 42 (11), 1009–1023. doi: 10.1016/j.it.2021.09.003 34629295 PMC8496891

[B105] Walz P. H. Chamorro M. F. Falkenberg M. S. Passler T. van der Meer F. Woolums A. R. (2020). Bovine viral diarrhea virus: An updated American college of veterinary internal medicine consensus statement with focus on virus biology, hosts, immunosuppression, and vaccination. J. Vet. Intern. Med. 34 (5), 1690–1706. doi: 10.1111/jvim.15816 32633084 PMC7517858

[B106] Walz P. H. Grooms D. L. Passler T. Ridpath J. F. Tremblay R. Step D. L.. (2010). Control of bovine viral diarrhea virus in ruminants. J. Vet. Intern. Med. 24 (3), 476–486. doi: 10.1111/j.1939-1676.2010.0502.x 20384958

[B107] Wang X. Li F. Han M. Jia S. Wang L. Qiao X.. (2020). Cloning, prokaryotic soluble expression, and analysis of antiviral activity of two novel feline IFN-omega proteins. Viruses 12 (3), 335. doi: 10.3390/v12030335 32204464 PMC7150924

[B108] Wang J. Liu B. Wang N. Lee Y. M. Liu C. Li K. (2011). TRIM56 is a virus- and interferon-inducible E3 ubiquitin ligase that restricts pestivirus infection. J. Virol. 85 (8), 3733–3745. doi: 10.1128/JVI.02546-10 21289118 PMC3126137

[B109] Xie B. Zhao M. Song D. Wu K. Yi L. Li W.. (2021). Induction of autophagy and suppression of type I IFN secretion by CSFV. Autophagy 17 (4), 925–947. doi: 10.1080/15548627.2020.1739445 32160078 PMC8078712

[B110] Xu C. Feng L. Chen P. Li A. Guo S. Jiao X.. (2020). Viperin inhibits classical swine fever virus replication by interacting with viral nonstructural 5A protein. J. Med. Virol. 92 (2), 149–160. doi: 10.1002/jmv.25595 31517388

[B111] Yesilbag K. Alpay G. Becher P. (2017). Variability and global distribution of subgenotypes of bovine viral diarrhea virus. Viruses 9 (6), 128. doi: 10.3390/v9060128 28587150 PMC5490805

[B112] Zhang L. Qin Y. Chen M. (2018). Viral strategies for triggering and manipulating mitophagy. Autophagy 14 (10), 1665–1673. doi: 10.1080/15548627.2018.1466014 29895192 PMC6135629

[B113] Zhang C. Wang X. Sun J. Guo M. Zhang X. Wu Y. (2021). Autophagy induced by the n-terminus of the classic swine fever virus nonstructural protein 5A protein promotes viral replication. Front. Microbiol. 12, 733385. doi: 10.3389/fmicb.2021.733385 34512612 PMC8424089

[B114] Zhou J. Chen J. Zhang X. M. Gao Z. C. Liu C. C. Zhang Y. A.. (2018). Porcine Mx1 protein inhibits classical swine fever virus replication by targeting nonstructural protein NS5B. J. Virol. 92 (7), e02147–e02117. doi: 10.1128/JVI.02147-17 29343573 PMC5972896

[B115] Zurcher C. Sauter K. S. Mathys V. Wyss F. Schweizer M. (2014a). Prolonged activity of the pestiviral RNase erns as an interferon antagonist after uptake by clathrin-mediated endocytosis. J. Virol. 88 (13), 7235–7243. doi: 10.1128/JVI.00672-14 24741078 PMC4054414

[B116] Zurcher C. Sauter K. S. Schweizer M. (2014b). Pestiviral e(rns) blocks TLR-3-dependent IFN synthesis by LL37 complexed RNA. Vet. Microbiol. 174 (3-4), 399–408. doi: 10.1016/j.vetmic.2014.09.028 25457366

